# Spatial frequency domain imaging using an analytical model for separation of surface and volume scattering

**DOI:** 10.1117/1.JBO.24.7.071604

**Published:** 2018-09-14

**Authors:** Steffen Nothelfer, Florian Bergmann, André Liemert, Dominik Reitzle, Alwin Kienle

**Affiliations:** Institut für Lasertechnologien in der Medizin und Meßtechnik, Ulm, Germany

**Keywords:** spatial frequency domain imaging, reduced scattering coefficient, absorption coefficient, radiative transfer equation, surface scattering, bidirectional reflectance distribution function

## Abstract

A method to correct for surface scattering in spatial frequency domain imaging (SFDI) is presented. The use of a modified analytical solution of the radiative transfer equation allows calculation of the reflectance and the phase of a rough semi-infinite geometry so that both spatial frequency domain reflectance and phase can be applied for precise retrieval of the bulk optical properties and the surface scattering. For validation of the method, phantoms with different surface roughness were produced. Contrarily, with the modified theory, it was possible to dramatically reduce systematic errors due to surface scattering. The evaluation of these measurements with the state-of-the-art theory and measuring modality, i.e., using crossed linear polarizers, reveals large errors in the determined optical properties, depending on the surface roughness, of up to ≈100%. These results were confirmed with SFDI measurements on a phantom that has a structured rough surface.

## 1 Introduction

Quantitative spatial frequency domain imaging (SFDI) for separation of the absorption ($\mu_{a}$) and the reduced scattering coefficient ($\mu_{s}^{\prime }$) gained importance in biomedical imaging over the last years. It facilitates the investigation of different kinds of tissue changes and biological processes, e.g., blood perfusion, neuronal death, and brain cellular composition changes.^1^^–^^3^ To eliminate specular and surface reflections in SFDI, crossed linear polarizing filters are generally used.^4^ However, as shown by Wiest et al.,^5^ the use of crossed polarizers causes significant errors in $\mu_{s}^{\prime }$ and $\mu_{a}$ when using a nonpolarized model for solving the inverse problem. As it is shown in this work, surface scattering $R_{S}$ leads to an offset in the spatial frequency resolved reflectance $R_{\mathrm{SFD}}(F)$. This offset mostly affects high spatial frequencies F, as for those $R_{\mathrm{SFD}}(F)$ is steadily decreasing^6^^,^^7^ and therefore, the ratio $\frac{R_{s}}{R_{\mathrm{SFD}}(F)}$ is increasing. Correcting for this offset without any more information than $R_{\mathrm{SFD}}(F)$ is hardly possible, because the subdiffusive scattering properties (e.g., phase function) and the surface scattering affect the spatial-frequency resolved reflectance in a similar way. Bassi et al.^8^ were the first to investigate the phase shift due to turbid media, but the phase information was not used for solving the inverse problem. By now, the phase information was only used for determining the topography of the sample in order to correct for changes of the reference intensity.^9^

First, results associated to this work were already presented on the European Conference on Biomedical Optics in 2017.^10^ However, the modified solution of the radiative transfer equation (RTE) is now presented in more detail and new results of measurements are shown. The modified solution makes it possible to correct for surface scattering by solving the inverse problem not only taking the reflectance $R_{\mathrm{SFD}}(F)$ but also the spatial shift or phase $\phi_{\mathrm{SFD}}(F)$ into account. It is shown that this phase is significantly influenced by surface scattering and thus appropriate for separating volume and surface scattering. The theoretical results were confirmed by SFDI measurements using specially prepared solid phantoms.

## 2 Theory

In order to model the light propagation in turbid media, an analytical solution of the RTE for semi-infinite media is applied within this work. The analytical solution of the RTE is based on a series expansion in rotated spherical harmonics of order M. A detailed derivation can be found in literature.^11^^,^^12^ The solution of this derivation gives the complex reflectance:

$$R_{\mathrm{med}}(q,n_{\mathrm{med}},n_{\mathrm{ext}},\mu_{a},\mu_{s}^{\prime },\rho_{V},\theta_{0}^{\prime },\theta_{\mathrm{NA}}),$$ (1)

originating from the volume scattered light. Note that the solution in Ref. 12 has its source inside the medium, this is why in case of an external source, it additionally has to be scaled by a transmission coefficient $T_{V}$. The reflectance depends on the angular spatial frequency q=2πF, the refractive indices of both the internal ($n_{\mathrm{med}}$) and external ($n_{\mathrm{ext}}$) medium, the absorption coefficient $\mu_{a}$, the scattering $\mu_{s}$, or alternatively, the reduced scattering coefficient $\mu_{s}^{\prime }$, the phase function $\rho_{V}$ of the scatterers and the angle:

$$\theta_{0}^{\prime }=\arcsin(\frac{n_{\mathrm{ext}}}{n_{\mathrm{med}}}\;\sin\;\theta_{0}),$$ (2)

which is the angle inside the medium calculated according to Snell’s law from the angle $\theta_{0}$ under which the light is irradiated relative to the normal of the sample surface. Moreover, the limited detection angle with respect to the numerical aperture of the objective lens is taken into account with $\mathrm{NA}=\sin(\theta_{\mathrm{NA}})$.

**Fig. 1:**
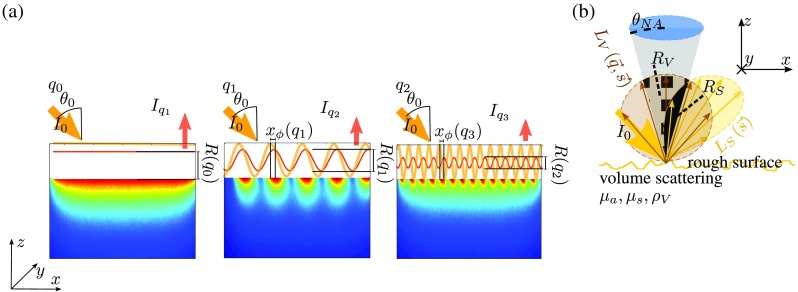
(a) Schematic illustration of the radiance inside a turbid medium, which is obliquely illuminated at $\theta_{0}$ by a sinusoidal intensity pattern. (b) Schematic illustration of the light being detected in a SFDI setup. A certain part $R_{V}$ of the detected light is coming from the volume (chessboard) and a second part $R_{S}$ is due to surface scattering (black solid), which both are the normalized integrals of the radiance $L_{V}$ and $L_{S}$, respectively.

The reflectance can be illustrated [compare Fig. 1(a)] as the amplitude of a sinusoidal illuminated pattern, which is being attenuated subject to the optical properties of the sample. For measurement of the reflectance, the sample is illuminated with a periodical intensity pattern, e.g., a collimated sinusoidal intensity of the kind:

$$I_{I}(x,y,z)=\frac{I_{0}}{2}[\sin(q_{i}x+\phi_{S}(q,z))+1],$$ (3)

with different spatial frequencies $q_{i}$. The additional phase $\phi_{S}(q,z)$ is due to the topography of the sample, which in detail is explained later in this section. The reflected intensity $I_{V,q_{i}}(x,y,z)$ due to volume scattering then yields

$$I_{V,q_{i}}(x,y,z)=\frac{I_{0}}{2}|T_{V}R_{\mathrm{med}}(q=q_{i})|\times \sin[q_{i}x+\arg(R_{\mathrm{med}}(q=q_{i}))+\phi_{S}(q=q_{i},z)]+\frac{I_{0}}{2}|T_{V}R_{\mathrm{med}}(q=0\;{\mathrm{mm}}^{-1})|,$$ (4)

where the transmission coefficient

$$T_{V}\equiv 1-{\overset{˜}{R}}_{S}$$

is the fraction of light that is not reflected at the surface but transmitted into the medium. The total surface reflection ${\tilde{R}}_{S}$ is defined as follows:

$${\overset{˜}{R}}_{S}\equiv |\frac{\int^{\Omega }L_{S}(\overset{\to }{s})d^{2}s}{\int^{4\pi }L_{I}(\overset{\to }{s})d^{2}s}|,$$ (5)

$$=\frac{\left|\int^{\Omega }L_{S}(\overset{\to }{s})d^{2}s\right|}{I_{0}},$$ (6)

which is the ratio of the integral over all directions of the surface reflected radiance $L_{S}$ and the integral over the half-space Ω of the incident radiance $L_{I}$. The transmitted light propagates through the turbid medium according to the RTE, whereby the modulation amplitude [thin red line in Fig. 1(a)] of the light being reflected from the sample can be expressed in terms of the analytical solution Eq. (1) of the RTE as follows:

$$R_{V}(q_{i})\equiv |\frac{\int^{\mathrm{NA}}L_{V}(q=q_{i},\overset{\to }{s})d^{2}s}{\int^{4\pi }L_{I}(\overset{\to }{s})d^{2}s}|,$$ (7)

$$R_{V}(q_{i})=|T_{V}R_{\mathrm{med}}(q=q_{i},n_{\mathrm{med}},n_{\mathrm{ext}},\mu_{a},\mu_{s}^{\prime },\rho ,\theta_{0},\theta_{\mathrm{NA}})|.$$ (8)

In this equation, the radiance $L_{V}$ is, as illustrated in Fig. 1(b), originated by light that was scattered in the turbid volume. However, besides the often investigated change in the amplitude $R_{V}$, scattering also leads to a significant spatial shift $x_{\phi }(q)$ of the detected sinusoidal intensity compared to the irradiated pattern.^8^ This phase shift shall be discussed shortly in order to understand the principle of separating volume and surface scattered light, which will be presented afterward. The reason for this shift is that the reflected light in average propagates through the medium by the effective free path before the first interaction with the medium takes place. The oblique irradiation then results in a projection of this mean free path to the x dimension, which corresponds to the measured spatial shift $x_{\phi }(q)$. The phase

$$\phi_{\mathrm{med}}(q)=\arg[R_{\mathrm{med}}(q,n_{\mathrm{med}},n_{\mathrm{ext}},\mu_{a},\mu_{s}^{\prime },\rho ,\theta_{0},\theta_{\mathrm{NA}})],$$ (9)

due to the volume scattering therefore depends on both the incident angle $\theta_{0}$ and the optical properties. However, experimentally, the phase shift always refers to a reference measurement, whereas the surface of the reference sample defines the position z=0. Any type of misalignment z≠0 of the sample surface then will cause an additional phase shift of

$$\phi_{S}(q,z)=\frac{zq}{\tan(90\;\deg-\theta_{0})}=z\;q\;\tan(\theta_{0}).$$ (10)

Experimentally, it is impossible to perfectly align the sample. For this reason, the distance z of misalignment has to be included into the model in order to correctly fit the phase. This is why the spatial shift illustrated in Fig. 1(a) in total reads

$$x_{\phi }=\frac{\phi_{V}(q)}{q},$$ (11)

with the definition:

$$\phi_{V}(q_{i})\equiv [\phi_{S}(q=q_{i})+\phi_{\mathrm{med}}(q=q_{i})].$$ (12)

If not stated otherwise, whenever the phase is fitted the misalignment, z is always used as additional fit parameter. But as this is not crucial for separation of volume and surface scattering, the misalignment will not be discussed any further within this work.

For samples with an optically smooth surface, which has a Scratch-Dig<80,^13^ the total reflection ${\tilde{R}}_{S}$ is given by Fresnel’s equation according to

$${\overset{˜}{R}}_{S,\text{Fresnel}}(\theta_{0},n_{\mathrm{med}},n_{\mathrm{ext}})=\frac{1}{2}{\left|\frac{n_{\mathrm{ext}}\;\cos(\theta_{0})-n_{\mathrm{med}}\;\cos(\theta_{0}^{\prime })}{n_{\mathrm{ext}}\;\cos(\theta_{0})+n_{\mathrm{med}}\;\cos(\theta_{0}^{\prime })}\right|}^{2}+\frac{1}{2}{\left|\frac{n_{\mathrm{med}}\;\cos(\theta_{0})-n_{\mathrm{ext}}\;\cos(\theta_{0}^{\prime })}{n_{\mathrm{med}}\;\cos(\theta_{0})+n_{\mathrm{ext}}\;\cos(\theta_{0}^{\prime })}\right|}^{2}$$ (13)

with

$$\sin\;\theta_{0}^{\prime }=\frac{n_{\mathrm{ext}}}{n_{\mathrm{med}}}\;\sin\;\theta_{0}.$$ (14)

This solution describes the light propagation in a semi-infinite turbid medium exact as long as the direct reflex does not fall into the aperture of the detection system.

For a large number of practically relevant cases, the requirement of an optically smooth surface is not fulfilled. In the case of rough surfaces, the detected light is always a superposition of light reflected from the turbid volume and light that is diffusely reflected at the surface. In order to describe the ratio of light, which is scattered at the surface into a certain direction $\vec{s}(\theta_{S},\phi_{S})$ with spherical coordinates $\theta_{S}$ and $\phi_{S}$, the so-called bidirectional reflectance distribution function (BRDF):

$$\rho_{\mathrm{BRDF},S}(\theta_{I},\phi_{I},\theta_{S},\phi_{S})=\frac{dL_{S}(\theta_{S},\phi_{S})}{L_{I}(\theta_{I},\phi_{I})\cos(\theta_{i})d\theta_{I}\;d\phi_{I}}$$ (15)

has to be introduced. The index S indicates that the light is only scattered by the surface and did not penetrate into the volume. This function describes the ratio between the incident radiance $L_{I}(\theta_{I},\phi_{I})$ and the differential radiance $dL_{S}(\theta_{S},\phi_{S})$ of surface scattered light being detected, where the incident direction $\vec{s}(\theta_{I},\phi_{I})$ is given by the polar angle $\theta_{I}$ and the azimuthal angle $\phi_{I}$.

A simple assumption that can be made for the angular distribution of the light scattered at the surface is a constant BRDF. However, as Kienle and Foschum^14^ showed such Lambert surfaces do not exist in practice, thus, more precise models have to be applied. For instance, Sun^15^ utilize a statistical model of the surface for approximating the angular surface scattering function.

However, in order to only correct for surface roughness when solving the inverse problem, the exact knowledge of the BRDF is even not necessary, as the detected intensity

$$I_{S,q_{i}}(x,y,z)=R_{S}\frac{I_{0}}{2}[\sin(q_{i}x+\phi_{S}(q_{i},z))+1],$$ (16)

due to surface scattered light only depends on the surface scattering correction parameter $R_{S}$. This parameter is according to Fig. 1(b) given by the integral:

$$R_{S}=\int_{0}^{2\pi }\int_{0}^{\theta_{NA}}\int_{0}^{2\pi }\int_{0}^{\pi }\rho_{\mathrm{BRDF},S}(\theta_{i},\phi_{i},\theta_{d},\phi_{d})\delta (\phi_{i})\delta (\theta_{i}-\theta_{0})\times \sin(\theta_{i})\sin(\theta_{d})d\phi_{i}\;d\theta_{i}\;d\phi_{d}\;d\theta_{d},$$ (17)

$$=\int_{0}^{2\pi }\int_{0}^{\theta_{\mathrm{NA}}}\rho_{\mathrm{BRDF},S}(\theta_{0},0,\theta_{d},\phi_{d})\sin(\theta_{d})d\phi_{d}\;d\theta_{d},$$ (18)

and therefore, the exact knowledge of the BRDF is not necessary. The intensity defined in Eq. (16) therefore can be seen as this fraction of surface scattered light, which falls into the aperture of the detection system. In the case of a rough surface with a certain BRDF, the total reflection parameter ${\tilde{R}}_{S}$ is no longer given by Fresnel’s equation for plane surfaces but instead reads

$${\overset{˜}{R}}_{S}=\int_{0}^{2\pi }\int_{0}^{\pi /2}\rho_{\mathrm{BRDF},S}(\theta_{0},0,\theta_{d},\phi_{d})\sin(\theta_{d})d\phi_{d}\;d\theta_{d}.$$ (19)

Within this work, the Lambert approximation $\rho_{\mathrm{BRDF},S}=\text{constant}$ was used, for which Eq. (17) together with Eq. (19) yields

$${\overset{˜}{R}}_{S}=\frac{R_{S}}{1-\cos(\theta_{\mathrm{NA}})}.$$ (20)

It is moreover assumed that the surface scattering can be treated as a small perturbation to the Fresnel equations, which means that the light transmitted both into and out of the sample is disturbed much less by the rough surface than by volume scattering. This assumption also implies that the surface BRDF has no spatial dependency and surface scattering can be treated as local problem. In physical manner, this assumption is fulfilled, if both, the RMS surface roughness $R_{q}$ and correlation length $l_{c}$ are much smaller than the mean free path $1/\mu_{s}$ of the volume scattering. It should also be mentioned that the model is restricted to statistical surfaces for which the Kirchhoff approximation (KA) is valid. According to Ulaby et al.,^16^ when considering a stationary isotropic Gaussian surface, the KA can be applied, if

kl>6

and

$$R_{q}<\frac{l_{c}^{2}}{2.76\lambda },$$

where λ is the electromagnetic wavelength and k=2π/λ the wave number.

In the case of a sinusoidal irradiation [compare Fig. 1(a)], the detected intensity $I_{q_{i}}=I_{V,q_{i}}+I_{S,q_{i}}$ can be understood as a superposition of surface and volume scattered light. For clarification, the difference between these two parts is schematically illustrated in Fig. 1(b), which shows the part of light that was diffusely reflected at the surface [black solid part in Fig. 1(b)] and light that entered the turbid medium and was then scattered back into the objective lens [chessboard in Fig. 1(b)]. The sum

$$I_{q_{i}}(x,y,z)=I_{V,q_{i}}+I_{S,q_{i}},$$ (21)

$$I_{q_{i}}(x,y,z)=\frac{I_{0}}{2}[R_{V}(q_{i})\sin(q_{i}x+\phi_{V}(q_{i}))+R_{V}(q=0\;{\mathrm{mm}}^{-1})],$$ (22)

$$+R_{S}\frac{I_{0}}{2}[\sin(q_{i}x+\phi_{S}(q_{i},z))+1],$$ (23)

of these two detected sine shaped intensities $I_{V,q_{i}}$ and $I_{S,q_{i}}$ again is a sine (see derivation in Appendix A):

$$I_{q_{i}}(x,y,z)=\frac{I_{0}}{2}R_{\mathrm{SFD}}(q=q_{i})\sin(q_{i}x+\phi_{\mathrm{SFD}}(q=q_{i},z))+\frac{I_{0}}{2}(R_{S}+R_{V}(q=0\;{\mathrm{mm}}^{-1})),$$ (24)

with the amplitude

$$R_{\mathrm{SFD}}(q=q_{i})=\sqrt{R_{S}^{2}+R_{V}^{2}+2R_{S}R_{V}\;\cos(\phi_{V}-\phi_{S})},$$ (25)

and phase

$$\phi_{\mathrm{SFD}}(q=q_{i},z)=\mathrm{arctan2}(R_{V}\;\cos(\phi_{V})+R_{S}\;\cos(\phi_{S}),R_{V}\;\sin(\phi_{V})+R_{S}\;\sin(\phi_{S})).$$ (26)

**Fig. 2:**
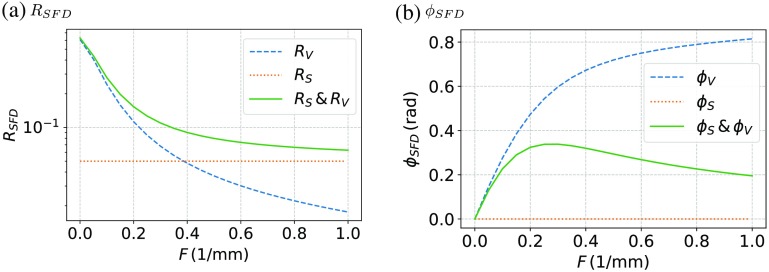
Theoretical calculations of (a) the reflectance $R_{SFD}$ and (b) the phase $\phi_{SFD}$ for different spatial frequencies F=q/2π and an irradiation angle of $\theta_{0}=45{}^{\circ}$ are shown. The optical properties are $\mu_{a}=0.01\;{\mathrm{mm}}^{-1}$, $\mu_{s}=4.00\;{\mathrm{mm}}^{-1}$, and $n_{\mathrm{med}}=1.4$, whereby a Henyey–Greenstein phase function with g=0.75 was utilized resulting in $\mu_{s}^{\prime }=1.00\;{\mathrm{mm}}^{-1}$. The blue dashed curve denoted with $R_{V}$ or rather $\phi_{V}$ shows the part which comes from volume scattering. The dashed blue line named $R_{S}$ or rather $\phi_{S}$ represents the surface scattered part, for which the surface scattering correction parameter $R_{S}=0.05$ was exemplarily chosen. The solid green line represents the totally detected reflectance $R_{SFD}$ composed of the two parts $R_{V}$ and $R_{S}$.

The amplitude measured for an angular spatial frequency $q_{i}$, thus, yields $\frac{I_{0}}{2}R_{\mathrm{SFD}}(q=q_{i})$, which in future is denoted as AC reflectance. The part $\frac{I_{0}}{2}(R_{S}+R_{V}(q=0\;{\mathrm{mm}}^{-1}))$ corresponds to the intensity, which would be measured utilizing an homogeneous illumination of intensity $I_{0}/2$, this is furthermore denoted as DC part. For modulated illuminations with $q\neq 0\;{\mathrm{mm}}^{-1}$, this DC part can be seen as the offset of the sine along the ordinate. According to Fig. 1(a), the total shift along x becomes $x_{\phi }=\phi_{\mathrm{SFD}}(q=q_{i},z)/q_{i}$, where $\phi_{\mathrm{SFD}}$ corresponds to the total measured phase shift, originating both from the turbidity of the medium and the surface reflection. As an example, Fig. 2 shows a calculation of the SFD reflectance $R_{\mathrm{SFD}}$ and phase $\phi_{\mathrm{SFD}}$ versus spatial frequency F=q/2π. The optical properties for this calculation were $\mu_{a}=0.01\;{\mathrm{mm}}^{-1}$, $\mu_{s}=4.00\;{\mathrm{mm}}^{-1}$, and $n_{\mathrm{med}}=1.4$, whereby a Henyey–Greenstein phase function with g=0.75 was utilized resulting in $\mu_{s}^{\prime }=1.00\;{\mathrm{mm}}^{-1}$. The total reflectance and phase are shown as green solid lines in Fig. 2 and those are composed of the part coming from the volume $R_{V}$ (blue dashed) and the surface $R_{S}$ (orange dotted). For this calculation, the surface scattering factor $R_{S}$ was exemplarily chosen to be 0.05, as for skin it is expected to obtain comparable values. The influence of surface scattering regarding $R_{\mathrm{SFD}}$ and the changes of the phase can clearly be seen.

In order to be able to neglect the influence of the phase function $\rho_{V}(\theta )$ on the determination of the other optical properties, the exact phase function of the used scatterers has to be taken into account. As the solution of the RTE is a series expansion in spherical harmonics, whereby in the following, M denotes the order of this expansion, the phase function likewise has to be expanded in terms of Legendre polynomials. This is why the solution of the transport equation takes i∈[0;M+1] expansion coefficients:

$$\rho_{i}=\int_{0}^{\pi }\rho (\theta )P_{i}(\cos(\theta ))\sin(\theta )d\theta ,$$ (27)

instead of the exact phase function. Though the accuracy of the solution depends on the expansion order, it was found that for an order of M≥9, these deviations do not significantly influence the results. Thus, within this work, all solutions of the RTE were calculated with an order of M=9.

## 3 Materials and Methods

All SFDI measurements within this work were carried out with the spatial frequency domain setup described by Bodenschatz et al.^17^ It consists of a tungsten polychromatic light source, a filter wheel equipped with eight bandpass color filters, a digital micromirror device (0.7 XGA VIS, Discovery 4100 Development Kit, Vialux, Germany), and a cooled charge coupled device (CCD) camera (QSI640, USA). The sinusoidal intensity patterns with possible spatial frequencies of $0\;{\mathrm{mm}}^{-1}\leq F\leq 1\;{\mathrm{mm}}^{-1}$ are projected under an oblique angle of $\theta_{0}=35\;\deg$ onto the sample and diffuse reflected light from a $38\;{\mathrm{mm}}^{2}\times 38\;{\mathrm{mm}}^{2}$ region is imaged by an objective lens (NA≈0.1) onto the CCD chip.

By projecting three sine patterns of J and different spatial frequencies $F_{i}$, each phase shifted by 0, 2π/3, and 4π/3, images with intensity values $I_{n,(u,v)}$ $\left(F_{i}\right)$ for n∈[1,2,3] at pixel (u,v) are acquired. Demodulating each pixel^18^^,^^19^ yields the DC intensity as follows:

$$I_{\mathrm{DC},(u,v)}(F_{i})=\frac{1}{3}(\overset{3}{\underset{n=1}{\sum }}I_{n,(u,v)}(F_{i})),$$ (28)

the AC intensities are

$$I_{\mathrm{AC},(u,v)}(F_{i})=\frac{\sqrt{2}}{3}\sqrt{\overset{3}{\underset{n=1}{\sum }}\overset{3}{\underset{k=1}{\sum }}{\left(I_{n,(u,v)}(F_{i})-I_{k,(u,v)}(F_{i})\right)}^{2}/2}\;\forall \;F_{i}\neq 0,$$ (29)

and the phase is calculated by

$$\phi_{\left(u,v\right)}(F_{i})=\mathrm{arctan2}(\overset{3}{\underset{n=1}{\sum }}I_{n,(u,v)}(F_{i})\sin(n2\pi /3),\times \overset{3}{\underset{n=1}{\sum }}I_{n,(u,v)}(F_{i})\cos(n2\pi /3))\;\forall \;F_{i}\neq 0.$$ (30)

Inhomogeneities in the intensity of the incident light field as well as the point spread function of the illumination system cause artifacts in the demodulated signal, this is why additionally, a reference phantom with known reflectance $R_{\mathrm{SFD},\mathrm{ref}}(F_{i})$ and phase $\phi_{\mathrm{SFD},\mathrm{ref}}(F_{i})$ has to be measured. With help of this reference measurement, the absolute SFD reflectance is

$$R_{\mathrm{SFD},(u,v)}(F)=\{\begin{array}{ll} \underset{i}{\sum }(\frac{I_{\mathrm{DC},(u,v)}(F_{i})}{I_{\mathrm{DC},\mathrm{ref}(u,v)}(F_{i})}R_{SFD,ref}(0))/J & F=0\\ \frac{I_{\mathrm{AC},(u,v)}(F_{i})}{I_{\mathrm{AC},\mathrm{ref},(u,v)}(F_{i})}R_{SFD,ref}(F_{i}) & F\in F_{i} \end{array},$$ (31)

and the SFD phase is calculated by

$$\phi_{\mathrm{SFD},(u,v)}(F)=\{\begin{array}{cc} 0 & F=0\\ \phi_{\left(u,v\right)}(F_{i})-\phi_{\mathrm{ref},(u,v)}(F_{i})+\phi_{\mathrm{SFD},\mathrm{ref}}(F_{i}) & F\in F_{i} \end{array}.$$ (32)

Note that hereinafter, omitting the declaration (u,v) indicates an averaging over a certain region of the image for which the error is given by the standard deviation of the mean value. All measurements were carried out with the SFDI setup described above at spatial frequencies $F_{1}=0.043\;{\mathrm{mm}}^{-1}$, $F_{2}=0.086\;{\mathrm{mm}}^{-1}$, $F_{3}=0.130\;{\mathrm{mm}}^{-1}$, $F_{4}=0.173\;{\mathrm{mm}}^{-1}$, $F_{5}=0.216\;{\mathrm{mm}}^{-1}$, and $F_{6}=0.432\;{\mathrm{mm}}^{-1}$. Afterward, the model introduced in Sec. 2 was fitted to the data using a nonlinear least squares DOGLEG^20^ algorithm, which was taken from the library CERES SOLVER.^21^ An example of such a fit is given in Sec. 4.1.

**Fig. 3:**
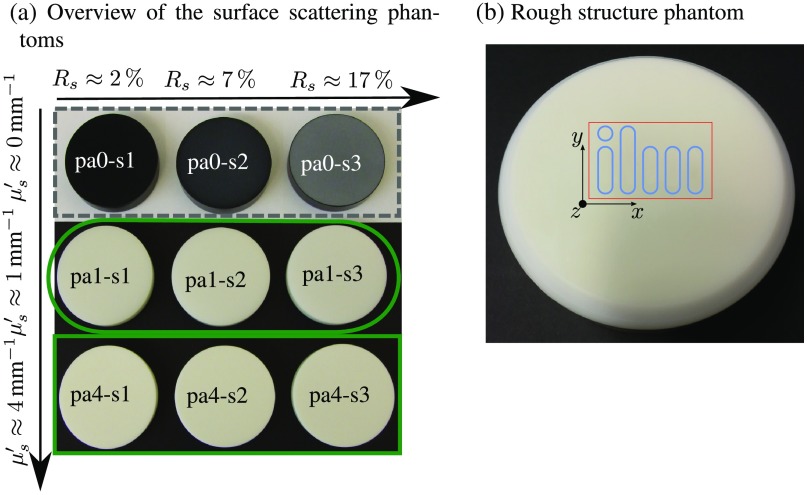
(a) Nine epoxy resin phantoms with different volume and surface scattering are shown. From top to bottom (pa0, pa1, pa4), the volume scattering increases from $\mu_{s}^{\prime }\approx 0\;\mathrm{mm}$ to $\mu_{s}^{\prime }\approx 4\;\mathrm{mm}$ and from left to right (s0, s1, s2), the surface roughness of the phantoms increases, as indicated by the surface scattering parameter $R_{s}$, which has been introduced in Sec. 2. This increase is clearly visible for the first row, for which the different shades of gray are only due to the various surface roughness. (b) A epoxy resin phantom, which contains a rough structure on its surface, is shown. The structure is almost invisible for the naked eye, this is why for convenience, the structure is additionally outlined in blue. The red box displays the region, for which the SFDI was done and the investigated model was fitted.

The utilized turbid samples (shown in Fig. 3) were all made from epoxy resin (Crystal Resin Giessharz glasklar, SuK Hock GmbH, Germany) with titanium dioxide added as scatterers. A precise description of the production process and a detailed characterization (e.g., absorption and scattering coefficient) of these kinds of solid-state phantoms was given by Krauter et al.^22^ Nine different phantoms were produced, from which three are nonturbid with $\mu_{s}^{\prime }\approx 0\;{\mathrm{mm}}^{-1}$ [Fig. 3(a), pa0], the next three have reduced scattering coefficients of $\mu_{s}^{\prime }\approx 1\;{\mathrm{mm}}^{-1}$ [Fig. 3(a), pa1] and another three have $\mu_{s}^{\prime }\approx 4\;{\mathrm{mm}}^{-1}$ [Fig. 3(a), pa4]. Within one group of phantoms with same reduced scattering (e.g., pa4), each of the phantoms were grinded with sand of different grain sizes resulting in three different surface roughnesses [Fig. 3(a), s1 to s3]. These surfaces were then analyzed with a contact profilometer (LV50E, Hommelwerke, Germany), which measures a topography profile y(x) along a line of $l_{x}=4.8\;\mathrm{mm}$ length by scanning a diamond stylus (tip radius 5  μm, tip angle 90 deg) in contact across the surface. Three of such profiles were measured at different positions for each of the phantoms pa0-s1, pa0-s2, and pa0-s3. The autocorrelations of the measured surface profiles revealed a Gaussian distributed profile for each of the surfaces; hence, the correlation length $l_{c}$ could be determined by fitting the normalized autocorrelation with the correlation model:

$$R_{c}(x)=\exp(\frac{x^{2}}{2l_{c}^{2}}).$$

According to the definitions given by the International Organization of Standardization (ISO), the so-called root mean square deviation $R_{q}$ and the maximum height of profile $R_{Z}$ were calculated from the surface profiles. These parameters are given in Table 1 for the three different surface roughnesses s1, s2, and s3. The values $R_{q}$ and $R_{Z}$ of the produced phantoms are within the range of values found in literature for skin. For example, Tchialeva et al.^23^ gave a summary of the skin surface roughness obtained with different optical methods, which yield a $R_{q}$ value between 0.1 and 10  μm.

**Table 1:** The table gives the root mean square parameter $R_{q}$ and maximum height of profile $R_{Z}$ calculated according to the ISO definition for the three different surfaces s1, s2, and s3 the measured surface profile. The table also shows the correlation length $l_{c}$, which could be determined form the autocorrelation of the profile, when considering a stationary isotropic Gaussian surface. The last column of the table shows that the KA is, according to Ulaby et al.,^16^ valid for all of the investigated surfaces.

Surface	$R_{q}$ (μm)	$R_{Z}$ (μm)	$l_{c}$ (μm)	KA
s1	0.203±0.009	1.53±0.06	12.5±0.5	Yes
s2	0.856±0.006	5.09±0.04	12.6±0.3	Yes
s3	3.01±0.03	16.5±0.16	12.9±0.4	Yes

Thus, a three times, three varieties of phantoms with different surface roughness (s1 to s3) and reduced scattering coefficient (pa0, pa1, and pa4) were investigated. No additional absorbers were added to the phantoms, hence, the absorption is only that of the pure epoxy resin $\left[\mu_{a}(600\;\mathrm{nm})\approx 10^{-3}\;{\mathrm{mm}}^{-1}\right]$, for which the absorption spectrum in detail is given by Krauter et al.^22^

Additionally, one phantom with homogeneous optical properties of $\mu_{s}^{\prime }\approx 1.05\;{\mathrm{mm}}^{-1}$ and $\mu_{a}\approx 0.44\;{\mathrm{mm}}^{-1}$ for the wavelength λ=700  nm was produced. The surface was then treated by sand blasting together with a template such that a certain region of the surface got rough and the remaining surface stayed smooth. Both the smooth and the rough surface were then characterized with the same contact profilometer mentioned before. This profile measurement revealed $R_{q}=(0.08\pm 0.02)\;\mu m$ and $R_{Z}=(1.4\pm 0.3)\;\mu m$ for the smooth region A, outlined in Fig. 6(a). The measurement of the rough region B, again displayed in Fig. 6(a), yielded $R_{q}=(1.8\pm 0.3)\;\mu m$ and $R_{Z}=(11.6\pm 0.5)\;\mu m$. A comparison of these values with the parameters given in Table 1 shows that the smooth and the rough surfaces are comparable with the surfaces s1 and s2, presented before. An image of this phantom is shown in Fig. 3(b) with blue lines surrounding the regions of the rough surface, which due to the volume scattering can hardly be seen by the naked eye. The red outlined box in Fig. 3(b) displays the region from which SFDI data were obtained and later investigated by means of the optical properties and surface roughness.

## 4 Results and Discussion

Two different sorts of phantoms were investigated within this work. In the following, the measurement results for these phantoms are presented and discussed.

### 4.1 Homogeneous Surface Roughness Phantoms

**Fig. 4:**
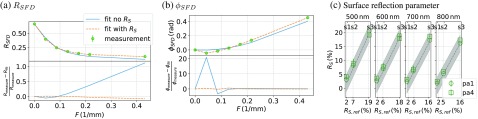
The green markers indicate the measured (a) SFD reflectance and (b) phase of the phantom pa1-s2 [see Fig. 3(a)], which were fitted by the model introduced in Sec. 2. The solid blue line shows the results for a fit without taking the surface scattering parameter $R_{S}$ into account, whereas the dashed orange line displays the results with $R_{S}$ being considered. The relative deviation between measured data and fitted model is given in the subplot at the bottom. The plots in panel (c) show the predicted against the known (from pa0) surface scattering parameter $R_{S}$ for four different wavelengths. The circular markers represent the fitted $R_{S}$ parameters for the group of low scattering phantoms pa1 ($\mu_{s}^{\prime }\approx 1\;{\mathrm{mm}}^{-1}$) and the quadratic markers the fitted surface scattering values for the group pa4 of phantoms with a reduced scattering coefficient of about $4\;{\mathrm{mm}}^{-1}$. The dashed gray line indicates the target values with a ±3% trust region, highlighted in brighter gray.

In this section, the results of the measurements with the phantoms shown in Fig. 3(a) are presented. SFD reflectance measurements of these phantoms, as described in Sec. 3, were carried out in order to obtain an averaged $R_{\mathrm{SFD}}$ and $\phi_{\mathrm{SFD}}$. The top plots in Figs. 4(a) and 4(b) exemplarily show the reflectance and phase for the phantom pa1-s2. The green markers indicate the measured data, which was fitted by two models, one which does not take surface scattering into account (blue solid line) and the new model that considers the surface scattering parameter $R_{S}$ (orange dashed line). The bottom plots in Figs. 4(a) and 4(b) show the relative deviation (measurement−fit)/measurement between measurement and fitted model respectively. It can be seen that the modified model fits the data far better than the model that does not contain the surface scattering parameter. The deviations for the model without $R_{S}$ are almost 1 for the reflectance and 20 for the phase. In comparison, the fit of the modified model, which takes $R_{S}$ into account, has a maximum deviation of 0.10 for the reflectance and 0.65 for the phase. This is more than a magnitude smaller for both, reflectance and phase, compared to a fit without surface scattering parameter $R_{S}$.

Fitting the modified theory yields besides the optical properties $\mu_{s}^{\prime }$ and $\mu_{a}$, the new parameter $R_{S}$, which as discussed in Sec. 2, is a measure of the surface scattering. In order to validate the correctness of $R_{S}$, SFD images of the three phantoms pa0-s1, pa0-s2, and pa0-s3 [compare Fig. 3(a), top row] were taken from which $R_{\mathrm{SFD}}$ could be demodulated according to Eq. (29) and as $R_{V}=0$ for these phantoms relation, Eq. (25) yields

$$R_{S}=R_{\mathrm{SFD}}.$$ (33)

These $R_{S}$ values were taken as reference and are denoted $R_{S,\mathrm{ref}}$. The trust region of ±3% for these values was estimated by multiple measurements of phantoms form group pa0. A validation of the correctness of $R_{S}$ after solving the inverse problem for the phantom groups pa1 and pa4 [compare Fig. 3(a) middle and bottom row], which both contain volume scattering, is given in Fig. 4(c). The green circles display the result for the phantom group pa1 against the reference values $R_{S,\mathrm{ref}}$ and the green squares indicate the results for the phantom group pa4, respectively. The dashed gray line indicates the target values with ±3% trust region, which is highlighted in brighter gray. The calculated $R_{S}$ values for both groups pa1 and pa4 are in average about 1% higher than expected, which is probably because of small variations in the measured reflectance values of the highest spatial frequency $F_{6}=0.432\;{\mathrm{mm}}^{-1}$. Theoretical investigations showed that especially high spatial frequencies are important for the correct separation of volume and surface scattering.

**Fig. 5:**
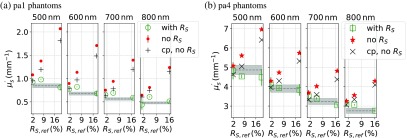
The plots in (a) and (b) show the fitted reduced scattering coefficients $\mu_{s}^{\prime }$ against the known surface scattering parameter $R_{S}$ evaluated with different models, whereas in panel (a), the results for the group pa1 of weakly volume scattering phantoms are displayed, and in panel (b), the results of the highly scattering phantoms pa4 are shown. Green circles and squares have been evaluated with the new model, which contains $R_{S}$. The red dots and red stars as well as the black crosses have been determined with a model, which does not take surface scattering into account. Note that the SFDI data for the black crosses were acquired with crossed linear polarizing filters between irradiation and detection. The dashed gray line indicates the known values with a trust region of ±5% relative deviation.

Solving the inverse problem without surface scattering implies a significant overestimation of the reduced scattering and absorption coefficient. This behavior is shown in Figs. 5(a) and 5(b) for each of the two phantom groups pa1 with $\mu_{s}^{\prime }\approx 1\;{\mathrm{mm}}^{-1}$ and pa4 with $\mu_{s}^{\prime }\approx 4\;{\mathrm{mm}}^{-1}$. The plots in Fig. 5 display $\mu_{s}^{\prime }$ against the different surface roughnesses s1, s2, and s3 quantized by the known surface scattering parameter $R_{S}$. The dashed gray lines indicate the known reduced scattering coefficients with a trust region of ±5% for different wavelengths. The trust region of $\mu_{s}^{\prime }$ was estimated by the production accuracy of the epoxy resin phantoms according to Krauter et al.^22^. The green circles and squares in Figs. 5(a) and 5(b) are the results obtained with the modified theory, which takes surface scattering into account. They all agree within $\pm 0.25\;{\mathrm{mm}}^{-1}$ with the reference, which is given as gray dashed line. The red dots and red stars in Figs. 5(a) and 5(b) indicate the values calculated without taking surface scattering into account. These values show a systematic increase of $\mu_{s}^{\prime }$ subject to surface scattering of the sample. The investigations show that without considering surface scattering in the utilized theory, the volume scattering is systematically overestimated.

In literature, e.g.,^9^^,^^24^^,^^25^ it is often stated that the use of crossed linear polarizers corrects for diffusely surface scattered light. Thus, in addition to the measurements with unpolarized light measurements with crossed linear polarizers between irradiation and detection were carried out both for the reference and the actual sample. The SFDI data obtained from these measurements were then evaluated with the solution of the unpolarized RTE. The black crosses in Figs. 5(a) and 5(b) mark $\mu_{s}^{\prime }$ found by solving the inverse problem. It appears that the crossed polarizers only reduce the influence of the surface scattered light a bit, but the overestimation of $\mu_{s}^{\prime }$ due to a rough surface is still present. Another method for reducing the surface scattering is to emerge the samples in a liquid (e.g., water), but as no analytical solution for a nonturbid layer on top of the actual scattering sample was available and moreover the surface scattering would still be present in a reduced kind, emerging the sample in a liquid was not further investigated within this work.

### 4.2 Structured Surface Roughness Phantom

**Fig. 6:**
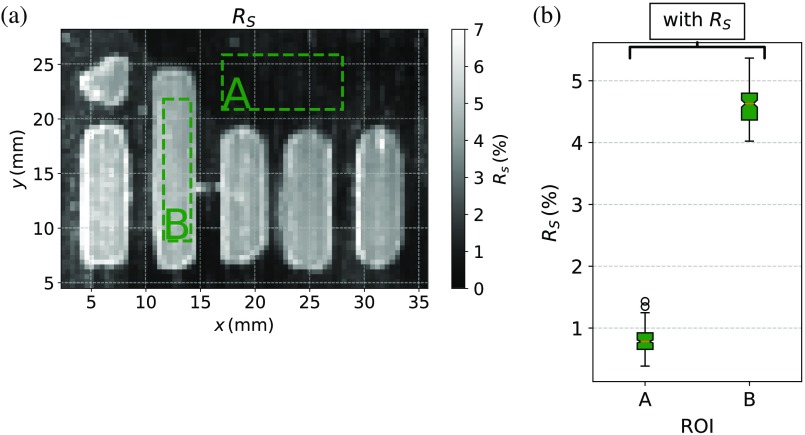
(a) The fitted $R_{S}$ parameter inside the red outlined region given in Fig. 3(b). is shown. (b) The box plot shows the averaged $R_{S}$ value of the outlined regions A and B.

In this part, the modified theory is applied for SFDI measurements of the spatially structured phantom shown in Fig. 3(b), which has both known scattering and absorption properties. Moreover, as already mentioned in Sec. 3, the glossy surface of the phantom has a structure with rough surface in the middle. After SFDI data were acquired as described in Sec. 3, the inverse problem was solved for both the modified theory which contains $R_{S}$ and a model, which does not correct for surface scattering. The calculated $R_{S}$ map for a wavelength of 700 nm can be seen in Fig. 6(a), for which the structured rough surface is clearly contrasted compared to the glossy surface surrounding the structure. A box plot of $R_{S}$ for the two outlined regions (A) and (B), glossy and rough, respectively, is given in Fig. 6(b). The values of the glossy surface A lie at around $R_{S}\approx 0.8\%$ and for the rough surface at about $R_{S}\approx 4.6\%$ and are, therefore, in the expected range. For the glossy surface, a roughness parameter of zero was striven for, but due to the hardening process of the initially liquid epoxy resin, an optical exactly smooth surface cannot be achieved. For this reason, the obtained surface scattering parameter $R_{S}$ for the glossy surface of the structured phantom seems reasonable. The rough surface was obtained by sand blasting with a grain size comparable to that of the particles utilized for grinding the phantoms with surface s2, which were discussed in the previous Sec. 4.1 and are shown in Fig. 3(a). The investigations discussed in the previous Sec. 4.1 yield $R_{S}\approx 5.5\%$ for such surfaces, which also agrees to the $R_{S}$ values obtained for the rough sandblasted structure.

**Fig. 7:**
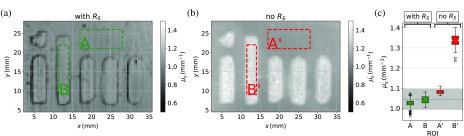
(a) Reduced scattering map calculated with the modified theory taking the $R_{S}$ parameter into account. The labeled region (A) is glossy, whereas region (B) has a sand blasted rough surface. (b) Reduced scattering calculated with the state-of-the-art method without taking surface scattering into account, again the regions labeled (A′) and (B′) indicate the same different surfaces as in (a). (c) Box plot of the averaged reduced scattering coefficients of the regions (A) and (B), where the surface scattering model was utilized compared to (A′) and (B′) for which the surface scattering was neglected.

Maps of the reduced scattering coefficient of the structured phantom are shown in Figs. 7(a) and 7(b), respectively. The inverse problem for the map Fig. 7(a) was calculated under consideration of $R_{S}$, whereas map Fig. 7(b) was determined without taking surface scattering into account. As the volume scattering and the reduced scattering coefficient of both regions should be the same, no structure should be present at all in Fig. 7(a). However, in both maps, the structure can clearly be seen, but compared to the map in Fig. 7(b), the reduced scattering coefficient in Fig. 7(a) is only different at the boundaries between smooth and rough surface. The evaluation of the SFDI data without $R_{S}$ yields $\mu_{s}^{\prime }$ values that are considerably different between glossy and rough surface. This finding is again stressed by the red markers denoted as “no $R_{S}$” in Fig. 7(c), which displays box plots of the outlined glossy region (A′) and the rough region (B′). Besides the systematically larger values compared to the reference (dashed gray line), a difference in $\mu_{s}^{\prime }$ of nearly $0.25\;{\mathrm{mm}}^{-1}$ between the two regions can be made out. By contrast, the evaluation with $R_{S}$, displayed in Fig. 7(a), only shows artifacts at the boundaries but the reduced scattering coefficients of glossy and rough surface areas are almost the same and agree with the reference values. These circumstances are again highlighted with boxplots displayed in Fig. 7(c). The green markers denoted at the top of the graph as “with $R_{S}$” indicate the averaged reduced scattering coefficient over the glossy region (A) and the rough area (B), both lie within the trust region of the known reference.

**Fig. 8:**
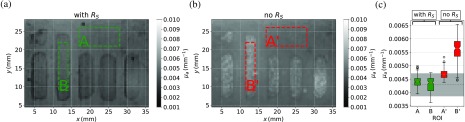
(a) Absorption map calculated with the modified theory taking the $R_{S}$ parameter into account. The labeled region (A) is glossy, whereas region (B) has a sand blasted rough surface. (b) Absorption map calculated with the state-of-the-art method without taking surface scattering into account, again the regions labeled (A′) and (B′) indicate the same different surfaces as in panel (a). (c) Box plot of the averaged absorption coefficients of the regions A and B, where the surface scattering model was utilized compared to (A′) and (B′) for which surface scattering was neglected.

The corresponding maps and box plots are shown for $\mu_{a}$ in Fig. 8. The contrast between glossy and rough surface for both evaluation methods “with $R_{S}$” and “no $R_{S}$” [compare Figs. 8(a) and 8(b)] is not as distinct as for $\mu_{s}^{\prime }$ but still present. The median absorption coefficients $\mu_{a}$ of the regions (A), (B), and (A′), (B′) charted in the boxplot [Fig. 8(c)] behave similar to $\mu_{s}^{\prime }$. Without $R_{S}$, the absorption in general is systematically too high and the difference between glossy (A′) and rough (B′) surface is approximately $\approx 0.001\;{\mathrm{mm}}^{-1}$.

## 5 Summary and Outlook

A modified solution of the RTE, which takes surface scattering into account, was presented. A parameter $R_{S}$ for quantifying surface scattering was introduced and the influence of this so-called surface scattering parameter on the determination of the optical properties was discussed. The modified theory was validated with different kinds of phantoms using SFDI experiments. These results, presented in Sec. 4, demonstrate significant improvements in the correctness and robustness of solving the inverse problem with the new model. Thus, a correction for systematic errors originated by diffuse surface reflections could be achieved without the need for crossed polarizers and consequently, a polarized light propagation theory.^5^ Since the surface roughness of the produced phantoms is comparable to that of skin, it is also demonstrated that surface roughness should be taken into account when applying SFDI to skin, e.g., for diagnosis.

In addition to the improved determination of the optical properties, the surface scattering parameter $R_{S}$ allows a quantification of the surface scattering. This could, for example, be used to differentiate between a lesion and surrounding healthy tissue, as the surface roughness in general is different.^26^

Currently, the Lambert approximation does not allow for quantifying the surface roughness with respect to parameters usually applied in metrology. In future work, a more precise surface BRDF model should be applied in order not only to determine a correction parameter but, for example, the RMS surface roughness $R_{q}$, an important parameter in surface metrology. This would allow the calculation of ISO roughness maps. However, for such evaluations, more experimental data are needed. This is why the setup has to be modified such that angularly resolved measurements at least at two different angles become possible.

## Figures and Tables

**Fig. 9:**
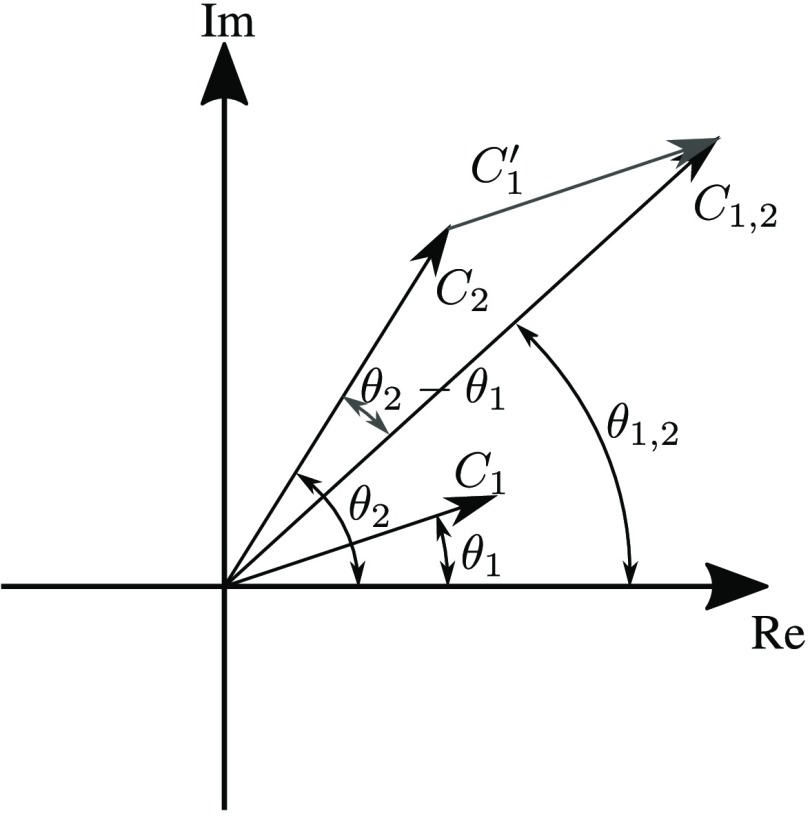
Schematic of the sum of two complex numbers $C_{1}=R_{1}\;\exp(i\theta_{1})$ and $C_{2}=R_{2}\;\exp(i\theta_{2})$ resulting in a complex number $C_{1,2}=C_{1}+C_{2}$.

## Appendix A: Sum of Two Sine Functions

It is to be shown that the sum of two sine functions $F_{1}=R_{1}\;\sin(\theta_{1})$ and $F_{2}=R_{2}\;\sin(\theta_{2})$ yields a sine function^27^ of kind $F_{1,2}=F_{1}+F_{2}=R_{1,2}\;\sin(\theta_{1,2})$ with

$$R_{1,2}=\sqrt{R_{1}+R_{2}+2R_{1}R_{2}\;\cos(\theta_{2}-\theta_{1})}$$ (34)

and

$$\theta_{1,2}=\mathrm{arctan2}(R_{1}\;\cos(\theta_{1})+R_{2}\;\cos(\theta_{2}),R_{1}\;\sin(\theta_{1})+R_{2}\;\sin(\theta_{2})).$$ (35)

Consider $R_{1}$, $R_{2}$ being the absolute values and $\theta_{1}$, $\theta_{2}$ the arguments of the two complex numbers $C_{1}=R_{1}\;\exp(i\theta_{1})$ and $C_{2}=R_{1}\;\exp(i\theta_{2})$, as shown in Fig. 9. The sum $C_{1,2}=C_{1}+C_{2}$ of these two complex numbers is just a sum of two vectors in the complex plane, for which the amount of $C_{1,2}$ is given by the law of cosine as follows:

$$R_{1,2}=\sqrt{C_{1}^{2}+C_{2}^{2}+2C_{1}C_{2}\;\cos(\theta_{2}-\theta_{1})}.$$ (36)

The angle between the real axis and $C_{1,2}$, which corresponds to the phase $\theta_{1,2}$, is coupled via the tangent with the imaginary:

$$I(C_{1,2})=I(C_{1})+I(C_{2}),$$ (37)

$$=R_{1}\;\sin(\theta_{1})+R_{2}\;\sin(\theta_{2}),$$ (38)

and real part

$$R(C_{1,2})=R(C_{1})+R(C_{2}),$$ (39)

$$=R_{1}\;\cos(\theta_{1})+R_{2}\;\cos(\theta_{2}),$$ (40)

of $C_{1,2}$ as

$$\tan(\theta_{1,2})=\frac{I(C_{1,2})}{R(C_{1,2})},$$ (41)

$$=\frac{R_{1}\;\sin(\theta_{1})+R_{2}\;\sin(\theta_{2})}{R_{1}\;\cos(\theta_{1})+R_{2}\;\cos(\theta_{2})}.$$ (42)

By applying the inverse of the tangent, the last expression yields as follows:

$$\theta_{1,2}=\mathrm{arctan2}(R_{1}\;\cos(\theta_{1})+R_{2}\;\cos(\theta_{2}),R_{1}\;\sin(\theta_{1})+R_{2}\;\sin(\theta_{2})).$$ (43)

As the imaginary parts of $C_{1}$, $C_{2}$, and $C_{1,2}$ exactly correspond to the sine functions Eqs. (34) and (35) are proven.
